# Supplementary material for: Genome-Wide Identification of the ABC Gene Family and Its Expression in Response to the Wood Degradation of Poplar in Trametes gibbosa
Source: J Fungi (Basel). 2024 Jan 24;10(2):96. doi: 10.3390/jof10020096 (PMC10889539; doi:10.3390/jof10020096)
Supplement: Supplementary file 1 [file jof-10-00096-s001.zip › Supplementary Materials.pdf]

Table S1. The DNA binding domain of *Tg-ABCs*

Description of Pfam	ID	The number of genes
ABC transporter	*gene_11539; gene_11540; gene_1251; gene_2690; gene_2753; gene_5243; gene_5257; gene_5291; gene_6080; gene_79; gene_8398; gene_9723*	12
ABC transporter transmembrane region	*gene_11539; gene_11540; gene_1251; gene_5243; gene_5291; gene_6080; gene_79; gene_8398; gene_9723*	9
ABC-2 type transporter	*gene_2690; gene_5257*	2
ABC-transporter N-terminal	*gene_2690; gene_5257*	2
CDR ABC transporter	*gene_2690; gene_5257*	2
ABC-2 family transporter protein	*gene_2753*	1

Table S2. Hydrophobicity of Tg-ABC proteins

ID	Number of amino acids	position of low score	amino acid of low score	low score	position of high score	amino acid of high score	high score
gene_11539	1536	415	Pro(P)	-2.556	136	Ala(A)	3.533
gene_11540	1497	1224	Lys(K)	-2.711	120	Leu(L)	3.500
gene_79	1459	178	Arg(R)	-2.989	351, 352	Leu(L), Ile(I)	3.433
gene_5291	1476	861	Arg(R)	-3.722	65	Leu(L)	3.211
gene_2753	1604	1595	Glu(E)	-3.500	348	Ile(I)	3.489
gene_2690	1465	817	Ser(S)	-3.056	764	Gly(G)	3.044
gene_6080	1463	596	Lys(K)	-3.322	343	Ala(A)	3.278
gene_5257	1524	38	Glu(E)	-2.944	660	Leu(L)	3.400
gene_5243	1685	657	Glu(E)	-3.678	31	Ala(A)	3.444
gene_1251	1622	862	Glu(E)	-3.000	337	Val(V)	3.700
gene_9723	1594	433	Lys(K)	-3.089	86	Ile(I)	3.144
gene_8398	1333	736	Asp(D)	-3.089	813	Ile(I)	3.033

Table S3. The result of the transmembrane helical segments analysis

ID	Number of predicted TMHs	Exp number of AAs in TMHs	Exp number, first 60 AAs	Total prob of N-in	outside	TMhelix	inside
gene_11539	16	364.050119999999	21.18813	0.01589	1-14,106-119,169-182,316-334,461-469,575-588,983-1004,1115-1185,1238-1536	15-37,83-105,120-139,146-168,183-205,296-315,335-357,438-460,470-492,552-574,589-608,960-982,1005-1027,1092-1114,1186-1208,1215-1237	38-82,140-145,206-295,358-437,493-551,609-959,1028-1091,1209-1214
gene_11540	16	355.513540000001	13.11442	0.99508	68-81,132-145,278-291,421-423,532-545,940-958,1053-1055,1165-1173	48-67,82-104,109-131,146-168,255-277,292-314,398-420,424-446,509-531,546-563,917-939,959-981,1030-1052,1056-1078,1142-1164,1174-1196	1-47,105-108,169-254,315-397,447-508,564-916,982-1029,1079-1141,1197-1497
gene_79	10	224.85683	0	0.12242	1-80,282-350,469-865,931-949,1008-1010,1118-1459	81-103,259-281,351-373,446-468,866-888,908-930,950-972,985-1007,1011-1030,1095-1117	104-258,374-445,889-907,973-984,1031-1094
gene_5291	12	264.577469999999	0.51167	0.99887	83-119,228-231,335-348,937-950,1054-1057,1166-1174	60-82, 120-142, 205-227, 232-254, 312-334, 349-371, 914-936, 951-973, 1031-1053, 1058-1080, 1143-1165, 1175-1197	1-59,143-204,255-311,372-913,974-1030,1081-1142,1198-1476
gene_2753	14	328.21852	21.93632	0.08149	49-227,294-302,354-357,423-844,1033-1066,1119-1127,1185-1214	26-48,228-250,271-293,303-325,334-353,358-380,400-422,845-867,1010-1032,1067-1089,1096-1118,1128-1150,1162-1184,1215-1237	1-25,251-270,326-333,381-399,868-1009,1090-1095,1151-1161,1238-1604
gene_2690	12	268.805920000001	0	0.02407	1-500,559-572,631-642,782-1172,1228-1246,1305-1318,1462-1465	501-523,536-558,573-595,608-630,643-665,759-781,1173-1192,1205-1227,1247-1269,1282-1304,1319-1341,1439-1461	524-535,596-607,666-758,1193-1204,1270-1281,1342-1438
gene_6080	10	227.190570000001	0.03763	0.04316	1-230,336-338,442-460,891-916,1007-1010,1118-1463	231-253,313-335,339-361,419-441,461-483,868-890,917-939,984-1006,1011-1030,1095-1117	254-312,362-418,484-867,940-983,1031-1094
gene_5257	12	265.04538	0	0.00040	1-542,601-626,675-683,819-1210,1269-1293,1346-1354,1499-1524	543-565,578-600,627-649,656-674,684-706,796-818,1211-1233,1246-1268,1294-1316,1323-1345,1355-1377,1476-1498	566-577,650-655,707-795,1234-1245,1317-1322,1378-1475

gene_5243	12	291.35256	21.41849	0.43787	1-14,165-183,307-325,452-454,563-1007,1124-1190,1309-1685	15-37,145-164,184-206,287-306,326-345,429-451,455-477,540-562,1008-1027,1101-1123,1191-1213,1286-1308	38-144,207-286,346-428,478-539,1028-1100,1214-1285
gene_1251	15	349.84945	11.97136	0.23896	137-150,203-216,358-371,549-551,676-1057,1110-1179,1243-1269,1322-1622	117-136,151-173,180-202,217-239,335-357,372-394,526-548,552-574,653-675,1058-1080,1087-1109,1180-1202,1223-1242,1270-1292,1299-1321	1-116,174-179,240-334,395-525,575-652,1081-1086,1203-1222,1293-1298
gene_9723	14	335.57323	22.31392	0.10665	1-4,101-104,160-173,336-349,497-1015,1079-1150,1214-1240,1290-1594	5-27,78-100,105-124,137-159,174-196,313-335,350-372,474-496,1016-1035,1056-1078,1151-1173,1194-1213,1241-1263,1270-1289	28-77,125-136,197-312,373-473,1036-1055,1174-1193,1264-1269
gene_8398	12	251.76514	0	0.88296	105-153,248-251,360-368,787-807,906-908,1011-1024	82-104,154-176,228-247,252-274,337-359,369-387,764-786,808-830,883-905,909-931,988-1010,1025-1047	1-81,177-227,275-336,388-763,831-882,932-987,1048-1333

Table S4. Prediction of phosphorylation site of Tg-ABC proteins

ID	Number of Phosphorylation site of Serine	phosphorylation site of Serine	Number of phosphorylation site of Threonine	phosphorylation site of Threonine	Number of phosphorylation site of Tyrosine	phosphorylation site of Tyrosine
gene_11539	123	7、24、49、51、58、59、75、90、98、101、111、113、131、144、145、148、152、212、227、232、260、268、293、298、305、306、309、311、330、348、360、363、385、388、389、395、404、417、430、434、456、467、486、552、557、581、585、586、595、613、645、659、674、676、677、709、713、731、734、748、752、784、802、817、818、858、882、885、911、954、979、995、998、1000、1004、1028、1032、1042、1057、1084、1089、1132、1143、1144、1147、1151、1165、1173、1195、1198、1212、1214、1215、1227、1228、1243、1275、1284、1286、1288、1289、1292、1299、1304、1316、1319、1320、1352、1365、1389、1394、1398、1401、1408、1418、1435、1457、1477、1511、1516、1524、1532、1536	92	32、36、47、57、82、114、126、130、158、172、173、174、194、261、263、277、281、290、299、303、316、331、346、349、371、387、396、436、474、500、528、554、562、566、571、579、608、624、631、640、658、661、668、671、675、707、712、754、792、800、810、814、835、853、864、876、889、945、953、956、971、973、991、1013、1046、1047、1054、1056、1064、1073、1111、1121、1134、1183、1187、1221、1229、1263、1314、1322、1331、1342、1355、1367、1382、1405、1446、1450、1467、1470、1497、1505	43	5、10、127、129、162、164、291、322、355、461、484、556、570、599、743、765、770、816、960、964、981、989、1001、1002、1006、1017、1024、1077、1155、1166、1172、1179、1207、1253、1271、1287、1336、1337、1445、1448、1495、1508、1534
gene_11540	126	4、9、23、30、38、39、44、53、61、65、69、94、100、111、115、139、182、185、190、218、220、253、265、266、271、281、290、312、323、345、351、355、362、369、386、418、423、442、498、500、513、537、541、542、569、587、592、593、595、598、606、615、629、634、638、666、670、688、691、705、709、741、759、774、775、815、833、839、842、855、859、868、882、911、930、934、936、950、952、955、961、985、989、994、998、999、1014、1030、1089、1100、1101、1104、1108、1122、1130、1155、1169、1171、1184、1200、1207、1238、1247、1251、1262、1267、1279、1282、1283、1287、1315、1328、1352、1357、1361、1364、	95	6、18、24、42、64、71、85、93、135、136、157、195、219、241、267、276、306、309、318、331、347、359、363、373、385、392、430、456、518、522、527、535、551、555、564、580、596、604、614、617、626、632、635、645、657、660、664、669、711、725、749、757、767、792、810、846、867、902、910、913、928、970、976、1003、1004、1011、1013、1021、1029、1041、1046、1091、1135、1144、1178、1186、1218、1226、1227、1277、1285、1294、1297、1305、1307、1318、1323、1330、1345、1368、1374、1407、1414、1431、1434	47	46、90、92、125、127、174、184、282、364、417、440、512、526、599、700、722、727、773、917、921、937、938、946、958、959、963、974、981、1034、1077、1112、1123、1129、1136、1161、1164、1196、1208、1216、1234、1250、1299、1300、1412、1459、1472、1492

		1371、1381、1398、1408、1421、1441、1475、1480、1496、1497				
gene_79	103	11、20、25、37、42、77、82、89、90、96、105、123、126、159、167、170、176、198、224、247、273、283、296、297、304、316、323、327、338、339、361、367、381、390、443、451、478、479、481、498、547、570、590、600、607、608、625、634、691、718、730、755、779、816、827、854、856、886、891、919、939、946、956、968、996、1001、1028、1033、1046、1055、1057、1064、1120、1136、1148、1156、1157、1166、1215、1235、1249、1251、1253、1260、1269、1272、1282、1287、1307、1312、1318、1321、1322、1340、1343、1346、1351、1354、1363、1374、1408、1444、1454	83	14、24、43、98、111、115、143、223、251、269、271、276、285、286、306、311、320、324、342、348、394、435、458、463、465、467、500、501、518、541、575、593、636、643、681、743、748、762、773、795、812、837、851、852、872、893、901、911、924、960、961、984、994、1006、1061、1089、1091、1108、1117、1132、1134、1135、1138、1145、1181、1190、1230、1236、1248、1284、1296、1325、1327、1329、1336、1370、1379、1386、1389、1395、1405、1425、1447	38	97、108、177、238、299、413、416、450、464、632、659、705、706、761、774、860、863、892、909、913、937、952、1011、1023、1025、1030、1049、1065、1077、1078、1133、1154、1165、1203、1295、1332、1409、1427
gene_5291	117	4、13、14、23、30、34、37、47、53、56、71、88、91、102、131、132、135、136、171、174、204、205、229、231、250、265、267、316、317、320、343、345、351、361、385、389、391、400、403、408、412、429、437、448、457、463、465、468、479、484、502、508、531、575、602、608、633、637、663、674、685、688、720、724、762、776、783、806、811、817、818、826、833、840、851、854、860、880、925、933、937、944、951、1008、1072、1077、1078、1103、1110、1147、1171、1185、1187、1190、1202、1216、1222、1235、1240、1242、1256、1270、1271、1273、1276、1290、1294、1302、1313、1316、1325、1338、1357、1374、1401、1469、1475	88	2、3、5、6、9、20、25、26、35、36、70、82、97、134、141、158、167、197、215、216、217、220、233、270、281、319、348、360、374、432、440、469、500、517、539、565、569、593、601、607、624、625、629、671、673、725、730、743、748、752、754、758、778、787、796、803、815、820、853、871、874、908、913、927、929、987、992、1028、1059、1130、1131、1142、1169、1178、1196、1206、1217、1219、1277、1303、1336、1364、1400、1414、1423、1424、1428、1453	24	83、154、214、397、435、478、494、499、563、823、941、956、1098、1099、1143、1151、1159、1170、1172、1243、1286、1332、1362、1454
gene_2753	127	21、73、87、90、108、115、121、131、141、171、186、220、241、247、258、268、273、277、279、319、321、331、339、354、357、389、391、435、438、441、456、464、475、488、491、505、511、515、533、600、617、627、633、642、643、684、687、698、	101	59、82、85、99、154、170、193、199、206、209、215、227、257、299、301、316、340、358、365、390、448、474、485、497、512、513、524、527、541、556、560、626、640、649、653、654、666、681、694、701、706、	40	38、55、70、153、173、221、242、282、373、427、429、558、594、693、729、740、764、769、887、984、1005、1032、1039、1106、

		705、709、731、732、733、739、765、767、768、770、775、800、801、805、807、826、829、845、860、891、903、908、927、932、946、951、956、980、999、1037、1046、1047、1054、1058、1072、1090、1114、1118、1123、1131、1156、1163、1170、1177、1182、1185、1190、1202、1203、1208、1233、1235、1249、1251、1259、1280、1281、1297、1305、1309、1348、1359、1371、1396、1406、1416、1419、1427、1437、1445、1475、1480、1501、1514、1529、1553、1562、1567、1578		710、723、730、763、774、798、803、809、820、833、848、868、873、877、880、901、919、920、922、933、937、941、958、966、971、986、991、1015、1080、1149、1151、1155、1160、1168、1196、1200、1201、1242、1250、1260、1275、1312、1325、1326、1327、1367、1374、1405、1446、1459、1472、1473、1498、1499、1508、1517、1542、1570、1575、1594		1115、1134、1142、1146、1152、1167、1210、1216、1220、1382、1389、1410、1506、1509、1523、1569
gene_2690	90	21、41、44、49、55、56、78、79、80、123、124、135、157、158、175、240、264、273、289、297、314、321、374、387、392、413、419、431、440、453、471、474、505、509、527、535、583、588、603、608、642、681、696、702、738、752、787、797、812、817、828、848、882、903、933、943、951、953、980、997、1000、1009、1015、1041、1042、1078、1086、1118、1134、1136、1169、1175、1184、1188、1190、1211、1229、1238、1239、1243、1259、1302、1311、1313、1352、1359、1363、1385、1412、1457	89	4、10、16、52、57、66、97、117、128、139、151、152、178、179、183、228、230、234、243、254、275、278、304、315、322、334、340、341、344、393、398、401、420、457、477、481、504、507、510、526、532、543、548、566、581、614、625、632、638、648、698、726、772、790、799、825、834、836、837、845、874、876、887、888、905、921、935、990、1008、1036、1047、1060、1135、1145、1149、1198、1212、1280、1286、1330、1335、1340、1355、1364、1372、1388、1398、1411、1453	62	76、114、134、191、200、212、218、279、346、353、369、379、385、396、444、476、520、521、568、571、595、600、615、620、657、660、675、676、682、715、742、747、779、783、792、827、851、867、869、923、945、960、1062、1076、1121、1147、1172、1203、1242、1245、1258、1266、1272、1338、1344、1349、1354、1403、1415、1418、1432、1462
gene_6080	113	9、18、48、50、53、58、65、69、88、108、125、129、131、147、148、153、157、166、182、189、210、249、250、253、262、270、279、283、291、300、310、337、372、382、419、420、433、436、454、455、475、487、505、562、616、626、629、630、649、650、658、713、721、728、738、740、757、806、814、858、860、885、901、903、923、927、931、935、938、956、971、974、985、993、996、997、1033、1034、1042、1046、1050、1053、1060、1067、	91	21、47、86、90、102、113、128、132、181、191、194、195、196、205、208、269、296、297、301、304、312、370、377、378、402、410、424、426、439、443、446、451、477、516、557、578、579、598、642、647、651、665、703、749、764、769、778、794、845、854、863、873、900、915、933、940、952、963、964、979、987、994、1001、1008、1055、1057、1061、1089、1121、1134、	45	98、163、211、213、234、256、336、389、390、396、440、468、499、654、727、745、780、782、795、812、862、865、912、916、1007、1011、1023、1024、1029、1030、1049、1052、1065、1086、1087、1133、1148、1163、1164、

		1075、1125、1132、1136、1142、1146、1182、1210、1230、1231、1244、1255、1273、1298、1320、1323、1326、1330、1333、1350、1360、1361、1379、1381、1422、1426、1434、1451、1455		1138、1140、1147、1165、1174、1175、1225、1279、1287、1289、1291、1310、1311、1322、1359、1366、1373、1382、1392、1412、1444		1198、1212、1252、1332、1395、1396
gene_5257	114	7、25、29、32、33、43、47、50、62、65、85、88、90、102、110、113、114、116、117、147、169、171、178、202、222、225、246、249、251、252、299、305、329、334、342、351、355、359、366、385、396、475、505、518、523、552、570、572、576、584、588、591、610、652、676、683、694、705、757、768、776、779、784、819、822、825、833、883、906、921、974、982、990、1019、1039、1048、1052、1054、1060、1068、1080、1157、1186、1187、1191、1252、1256、1260、1281、1285、1292、1305、1306、1360、1364、1377、1389、1411、1422、1427、1435、1448、1457、1465、1471、1493、1502、1504、1508、1511、1520、1521、1523、1524	111	16、18、60、61、83、98、99、120、175、197、226、230、275、277、281、287、288、290、313、323、360、367、379、386、437、438、446、464、500、522、562、571、573、592、625、632、635、665、667、669、695、702、719、738、759、764、765、774、778、788、808、814、818、824、834、836、866、875、879、915、926、927、936、942、960、968、972、1002、1028、1029、1047、1059、1075、1099、1110、1111、1143、1145、1146、1163、1164、1172、1183、1185、1190、1197、1198、1212、1231、1237、1238、1242、1253、1287、1291、1315、1320、1326、1339、1340、1368、1392、1412、1417、1425、1442、1447、1454、1458、1464、1495	54	51、238、247、259、265、391、398、408、414、424、430、457、471、488、502、520、615、644、701、704、710、726、753、780、785、787、789、889、908、962、999、1012、1101、1115、1133、1194、1213、1284、1308、1313、1327、1337、1338、1376、1386、1391、1393、1429、1433、1440、1452、1470、1492、1496
gene_5243	125	8、10、13、36、66、71、88、91、95、105、124、167、206、219、231、254、256、261、269、271、284、297、314、338、339、362、367、379、428、432、448、458、480、488、539、543、554、556、569、600、608、621、631、632、636、664、668、669、674、683、684、694、698、703、705、739、756、762、766、778、783、790、836、838、865、869、927、991、993、1006、1007、1042、1053、1087、1088、1091、1113、1114、1120、1133、1165、1174、1178、1185、1192、1233、1237、1244、1303、1308、1322、1325、1329、1336、1346、1366、1375、1376、1378、1383、1406、1423、1426、1431、1440、1460、1473、1487、1504、1508、1512、1516、1520、1535、1537、1539、1545、1556、1563、1580、1590、1591、1611、	104	15、38、53、84、89、113、125、138、140、177、181、182、195、217、224、225、243、246、258、272、282、292、294、358、397、430、461、469、489、547、572、575、609、622、645、699、710、722、731、734、737、742、753、754、765、828、855、871、889、894、917、920、966、1001、1010、1021、1047、1051、1127、1143、1148、1155、1170、1173、1195、1224、1235、1252、1273、1274、1279、1283、1290、1302、1321、1327、1338、1377、1379、1421、1427、1439、1452、1463、1470、1475、1502、1507、1511、1532、1538、1541、1543、1547、1550、1553、1560、1589、1596、1603、1615、1622、1642、1663	42	104、128、165、168、170、223、244、302、331、369、435、438、452、561、610、779、799、806、852、857、906、999、1002、1008、1039、1052、1057、1101、1103、1107、1126、1214、1221、1267、1278、1310、1331、1353、1372、1394、1496、1626

		1656、1661				
gene_1251	134	4、14、25、27、56、66、76、81、93、102、105、122、130、146、147、149、164、170、173、178、182、194、238、261、264、266、305、349、376、386、415、422、424、425、427、431、434、444、455、457、464、475、478、498、499、502、551、571、594、596、604、606、633、682、686、687、689、703、714、732、738、756、763、769、803、807、825、828、842、878、896、911、912、930、959、967、976、981、991、1039、1048、1049、1055、1061、1105、1116、1117、1127、1130、1131、1142、1184、1188、1195、1211、1217、1236、1250、1272、1294、1304、1312、1313、1328、1331、1347、1360、1362、1364、1369、1384、1401、1404、1405、1429、1437、1450、1479、1493、1496、1497、1500、1503、1520、1530、1543、1583、1585、1602、1607、1617、1618、1620、1621	100	9、24、43、45、53、61、87、123、133、140、161、192、206、208、228、259、269、330、346、362、367、396、397、403、408、429、430、435、439、446、468、473、476、479、491、514、515、518、578、584、592、663、667、670、672、680、719、725、747、761、771、780、801、806、821、848、886、894、904、929、947、952、970、979、1019、1071、1077、1089、1100、1119、1123、1132、1139、1141、1146、1149、1164、1170、1182、1235、1263、1301、1340、1348、1373、1399、1407、1416、1427、1440、1452、1461、1484、1490、1529、1536、1538、1553、1556、1563	57	13、29、48、51、75、145、160、162、183、196、198、242、268、279、295、297、332、354、359、391、392、532、545、568、641、645、657、671、818、837、859、864、869、910、1046、1078、1082、1087、1091、1102、1109、1205、1240、1251、1257、1264、1265、1289、1292、1338、1356、1372、1421、1422、1463、1534、1609
gene_9723	129	19、81、94、98、111、127、133、143、161、168、172、173、190、207、208、212、221、234、242、254、256、291、315、322、364、377、382、393、407、408、411、417、441、442、458、478、483、488、546、579、587、600、602、607、614、639、653、669、682、683、687、690、711、719、722、744、751、774、781、808、819、829、837、844、847、856、885、909、917、918、927、953、959、964、967、968、1021、1032、1044、1046、1050、1057、1068、1072、1087、1110、1127、1128、1136、1142、1151、1174、1180、1188、1192、1196、1214、1215、1229、1243、1250、1251、1264、1270、1280、1281、1299、1310、1313、1327、1331、1335、1372、1375、1380、1400、1409、1422、1427、1456、1459、1472、1475、1482、1499、1510、1530、1567、1571	98	18、37、53、54、92、120、131、146、150、154、156、182、196、215、220、231、244、245、253、272、273、298、313、367、368、372、380、381、399、415、420、424、448、451、452、466、496、567、584、592、606、610、612、629、660、662、671、673、679、691、702、736、862、880、911、923、939、955、960、963、966、969、973、974、978、1007、1008、1051、1073、1089、1108、1144、1145、1146、1149、1179、1190、1201、1206、1227、1257、1370、1376、1388、1405、1412、1419、1424、1428、1483、1508、1515、1522、1531、1534、1541、1569、1580	50	4、71、110、119、181、184、187、211、223、243、304、329、333、341、482、561、566、646、770、792、797、843、867、897、908、912、1009、1016、1018、1047、1058、1059、1063、1080、1091、1139、1169、1170、1176、1195、1198、1217、1232、1233、1343、1435、1441、1513、1545、1574

gene_8398	102	4、13、14、17、22、30、33、44、51、69、77、80、95、103、134、148、154、225、249、257、258、275、281、297、322、335、338、380、382、412、415、417、441、452、467、469、472、476、506、512、539、558、561、579、594、606、612、637、656、693、698、721、724、727、745、768、793、797、798、818、831、832、842、844、868、871、891、893、899、922、945、961、969、973、975、983、990、995、997、1013、1022、1032、1036、1045、1051、1052、1058、1066、1074、1084、1124、1129、1169、1214、1228、1231、1258、1262、1264、1289、1308、1331	77	27、40、58、71、78、110、119、167、169、177、211、215、217、240、276、290、298、301、307、356、357、394、406、431、454、460、461、473、497、516、520、521、533、569、605、610、628、629、631、638、658、687、695、735、777、796、837、863、867、869、884、894、917、958、963、1001、1023、1033、1034、1075、1098、1116、1130、1131、1145、1154、1173、1178、1188、1195、1201、1221、1257、1280、1281、1290、1310	39	155、158、168、170、176、189、246、277、315、345、348、404、439、482、567、646、670、744、747、748、755、764、778、827、925、972、989、1011、1096、1117、1139、1147、1156、1161、1175、1298、1321、1322、1324

Table S5. The result of the topological heterogeneity model prediction

ID	Number of N-glycosylation sites	Number of transmembrane helical segments
gene_11539	7	17
gene_11540	15	16
gene_79	6	10
gene_5291	9	12
gene_2753	16	14
gene_2690	3	12
gene_6080	9	12
gene_5257	9	12
gene_5243	10	13
gene_1251	9	15
gene_9723	8	13
gene_8398	4	12

Table S6. Primers sequence for quantitative real-time PCR

ID	Type	5' primers	3' primers
gene_11539	ABCC	GCTGCTCTTGATGTCCATACTG	ACTTCGCTTGCCACCTTGA
gene_11540	ABCC	GCTTCGCTCCAATATCACTATCA	ACTGTCTTCGCTCTCCTGTAG
gene_79	ABCC	TCTCCTCGCTCTCGCTCTT	TGACTGCTTCTCCACCTTCTC
gene_5291	ABCB	CCTGTTCTCCTTCCTCCTCTC	CATCCTTCACGAGCACATTCA
gene_2753	ABCA	CTGTGTATCCTGCGTTCTTCTC	ATGAGCGTAGCGTTGATTAGTG
gene_2690	ABCG	CGAGATGGCGAAGCGTTAC	AAGTGTGAGCGGTGTGAGAT
gene_6080	ABCC	GAGGCGAAGACGGAGAAGAG	GCTGCTGTGGATGAGAAGGT
gene_5257	ABCG	GGTGGTGGCAGTGGATGTA	GGCTGTATGGTGACGAGTTC
gene_5243	ABCC	CCGCCTTCCGTTGAATGTG	CTTGTCCTTGTCCTCCTCCTT
gene_1251	ABCC	CGGTATGGTGGCTATGATTCTG	ATGGACTTGCTCTCGGTAACA
gene_9723	ABCC	TGGTTGCGGATCTGGTCTG	CGTGATGGAGGCTTGAATGC
gene_8398	ABCB	GGTTCTTCATCGTCGCCATC	GGTGTTGTTCTCGTCCTTGTC
Gpd		AACGGTTTCGGTCGTATCGG	CTTGCCCTCGACCCAGAGCT

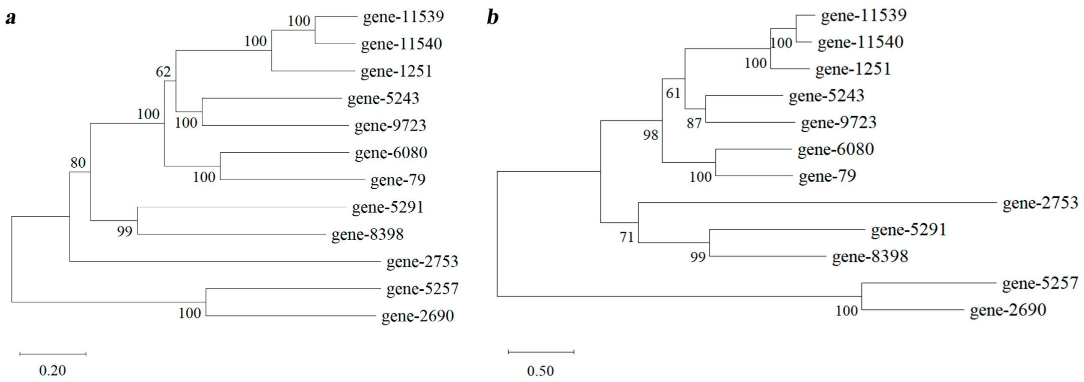

Figure S1. NJ-phylogenetic and ML-phylogenetic tree. (a) NJ-phylogenetic tree; (b) ML-phylogenetic tree

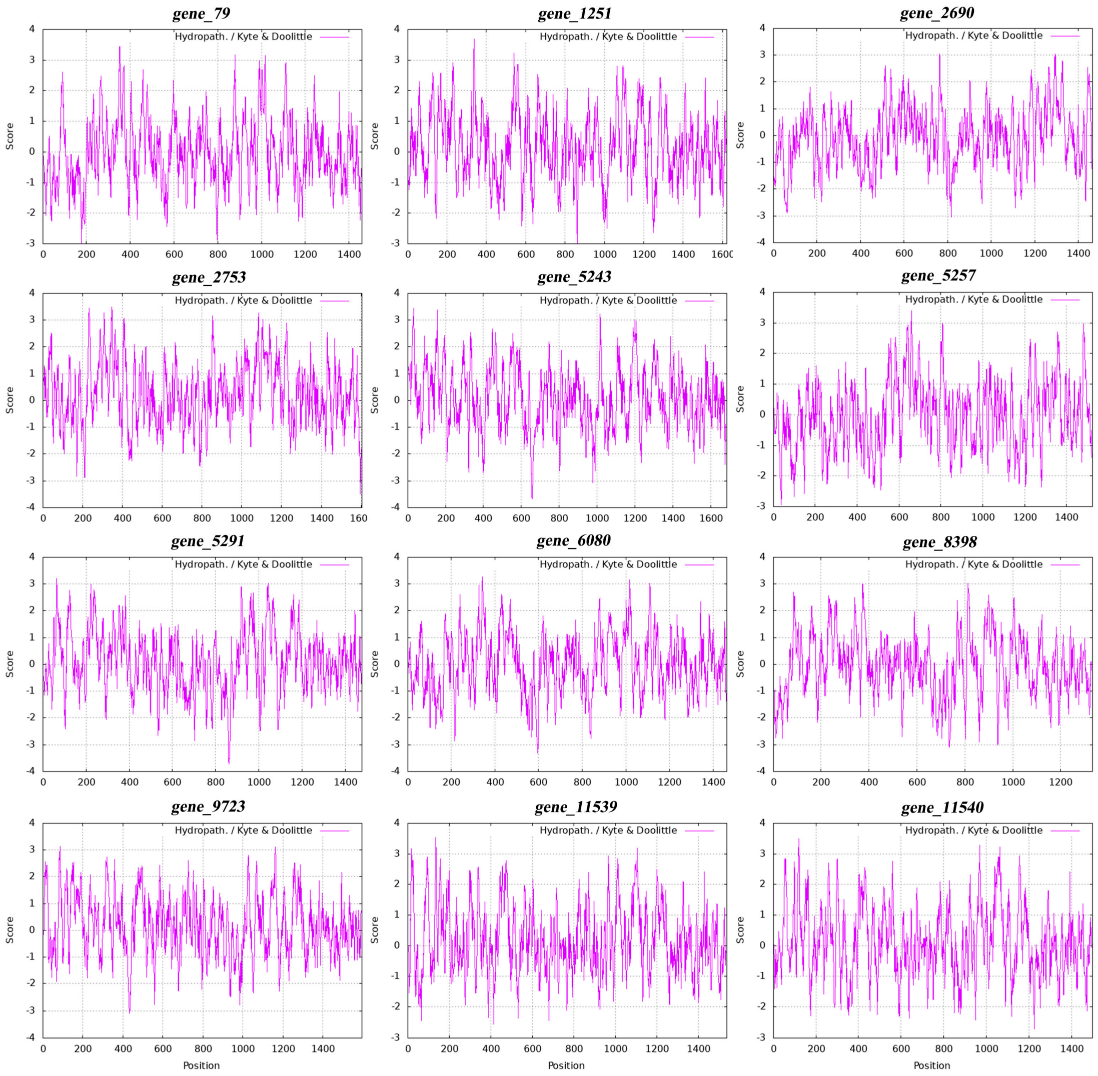

Figure S2. Hydrophobicity of Tg-ABC proteins

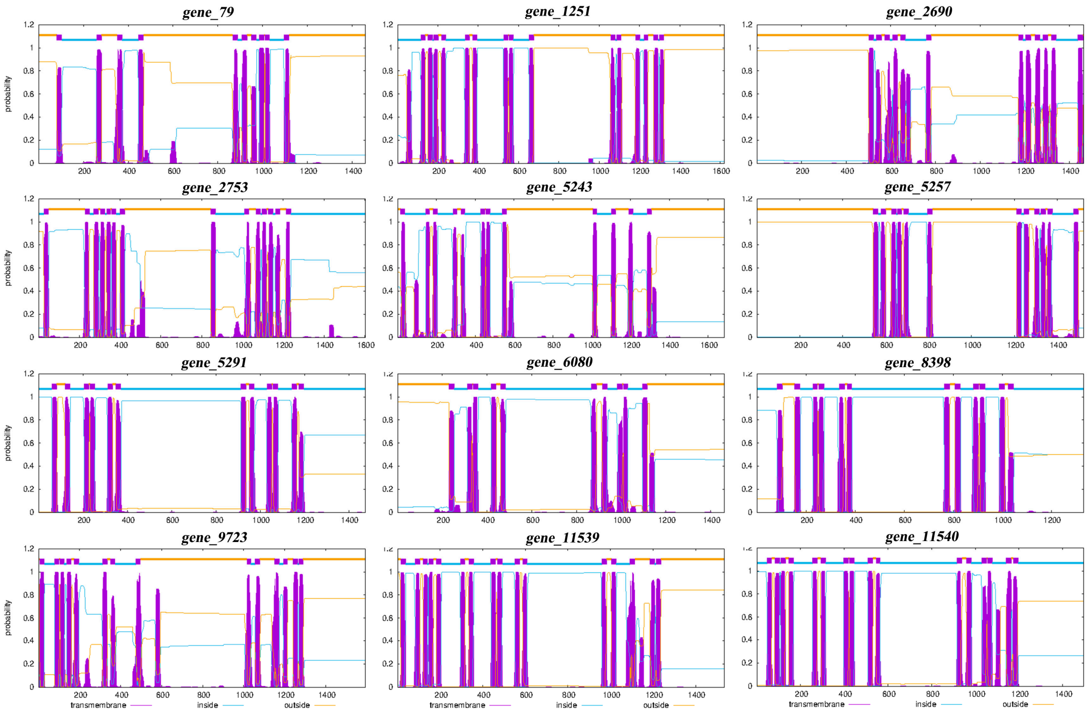

Figure S3. Transmembrane helical segments analysis

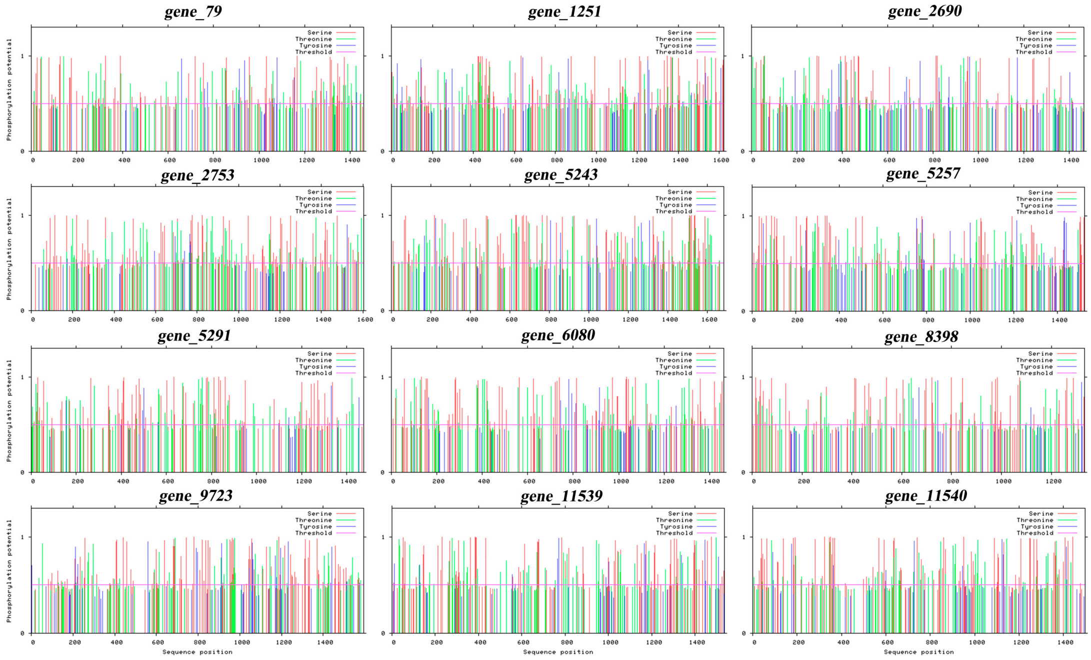

Figure S4. Phosphorylation site prediction

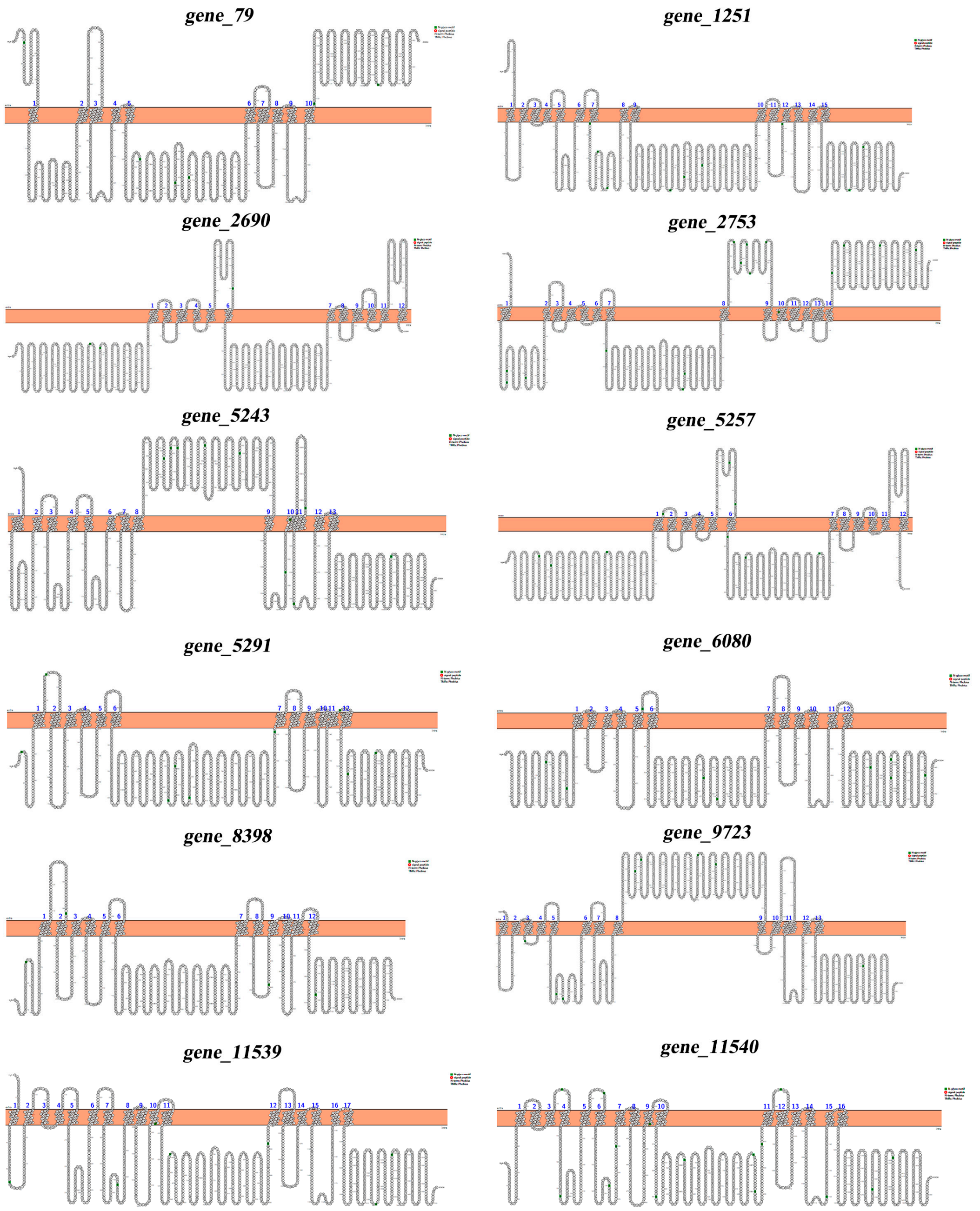

Figure S5. Topological heterogeneity model prediction
